# The evolving roles of extracellular vesicles in embryo-maternal communication

**DOI:** 10.1038/s42003-024-06442-9

**Published:** 2024-06-21

**Authors:** Alireza Fazeli, Kasun Godakumara

**Affiliations:** 1 Institute of Veterinary Medicine and Animal Sciences, Estonian University of Life Sciences, Tartu, Estonia; 2 Department of Pathophysiology, Institute of Biomedicine and Translational Medicine, Faculty of Medicine, Tartu University, Tartu, Estonia; 3 Division of Clinical Medicine, School of Medicine & Population Health, University of Sheffield, Sheffield, UK

**Keywords:** Embryology, Extracellular signalling molecules, Endocytosis

## Abstract

Mammalian reproduction relies on precise maternal-fetal communication, wherein immune modifications foster tolerance toward the semi-allogeneic embryo. Extracellular vesicles (EVs), including exosomes and microvesicles, have emerged as crucial mediators, transporting molecules like microRNAs securely. EVs influence various reproductive stages, from gamete maturation to implantation, and impact pathologies like pregnancy loss. In the embryo-maternal dialogue, EVs notably affect oviductal interactions, gene expression, and the embryo-endometrial interface, crucial for successful implantation. Key queries persist about EV uptake, cargo delivery, and the specific biomolecules driving communication. Their potential in diagnostics, therapeutics, and understanding environmental impacts on fertility signals an exciting future, reliant on collaborative efforts for transformative strides in reproductive health.

## Abstract (web summary)

Mammalian reproduction hinges on maternal-fetal communication mediated by extracellular vesicles. Their role in diagnostics and therapeutics shows promise, but questions about uptake, cargo delivery, and specific biomolecules remain.

## Introduction

In the realm of mammalian reproduction, pivotal events including gamete production, gamete maturation, fertilization, embryo development, implantation, and fetal growth occur within meticulously regulated parameters. Central to this orchestration is the intricate communication between maternal tissues and gametes/embryo, a mechanism critical in modulating the peri-implantation microenvironment to facilitate successful pregnancies^1^.

A crucial aspect of this communication involves immune modification, a process that is remarkable given the semi-allogeneic nature of the embryo, housing antigens transcribed from the paternal genome. Logically, the maternal immune system should reject this foreign entity. However, in a unique case of acquired immune tolerance, not only does the maternal immune system overlook the embryo, but it also actively supports implantation and, in certain species, subsequent invasion. These phenomena are believed to be instigated by embryo-maternal communication^2^.

**Fig. 1:**
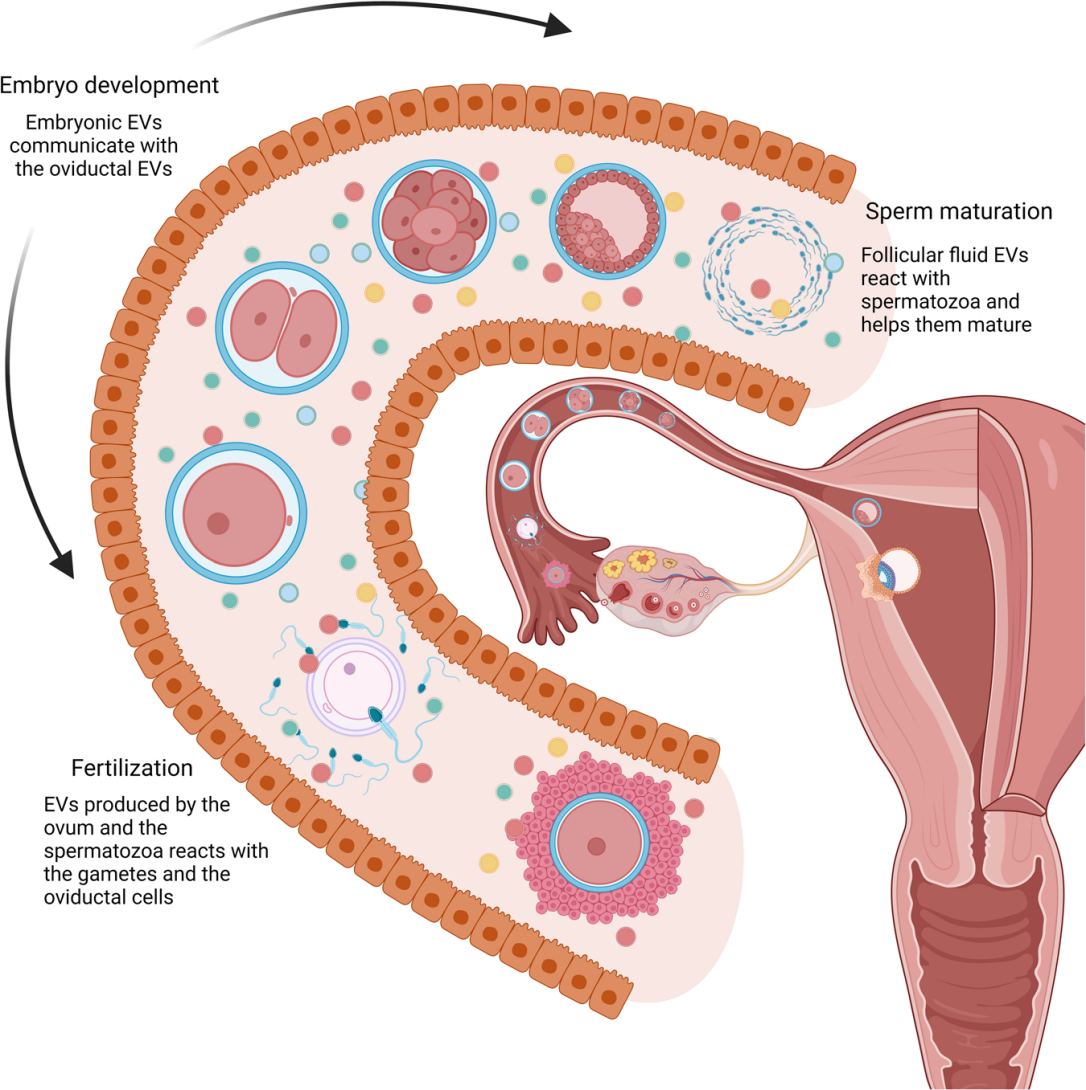
Influence of EVs in each step of the human reproduction process. Communication between gametes and the maternal system and the communications between the developing embryo and the maternal system are mediated by EVs in each step of the way.

Traditionally, embryo-maternal crosstalk has been attributed to endocrine, paracrine, or juxtacrine mechanisms involving diverse hormones and chemical signals produced by both the embryo and maternal tissue. Despite extensive research detailing various signaling pathways utilized in embryo-maternal communication, the comprehensive mechanism remains incompletely elucidated. Recently, intercellular signaling mediated by EVs has emerged as a novel facet of embryo-maternal dialogue^3–6^. The capacity of EVs to transport labile molecules, particularly microRNA (miRNA), within a secure encapsulated system, is hypothesized to be a crucial component of EV-mediated intercellular communication (Fig. 1).

## Extracellular vesicles

**Fig. 2:**
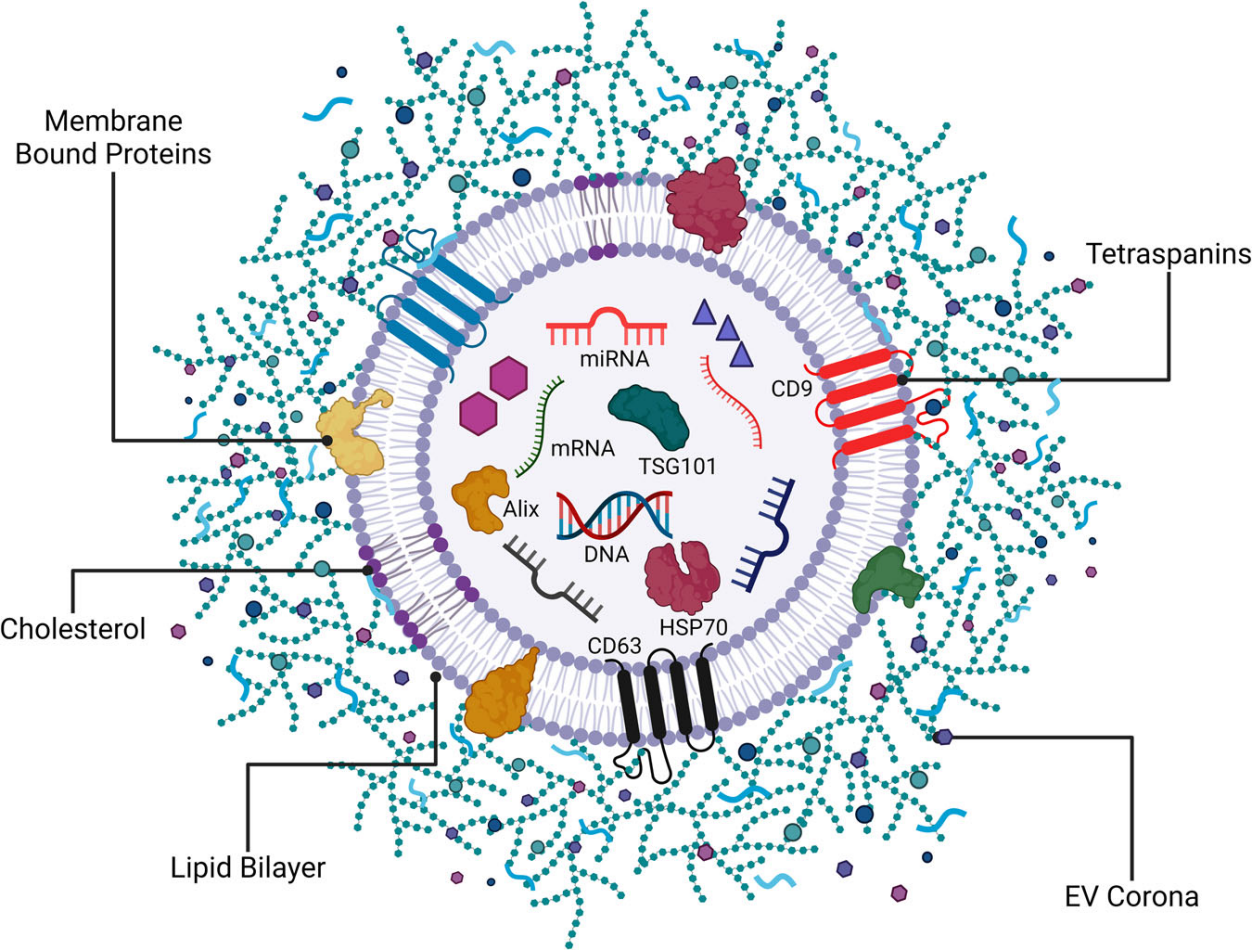
Morphology of a Typical EV. EVs are nanoscale membrane bound biological particles. Various biomolecules are contained within the EVs, bound to the Ev membrane, and loosely associated with the EVs in a corona.

EVs are nano-sized membrane-bound structures produced by diverse cell types through various biogenic processes^7^. They are broadly categorized into exosomes (40–100 nm), microvesicles (100–1000 nm), and apoptotic bodies (1–2 µm)^8,9^.Comprising lipids, proteins, RNAs (including lncRNA, mRNA, small non-coding RNA, rRNA, and miRNA), and DNAs (such as dsDNA, ssDNA, and mtDNA), EV composition and concentration are contingent upon physiological and environmental conditions (Fig. 2). EVs are influential regulators of different physiological and pathological conditions, inducing epigenetic and phenotypic changes in recipient cells^10,11^ and participating in various biological activities. They have potential applications as biomarkers for health and disease and as therapeutic targets^12^ .

In the realm of reproduction, EVs play diverse roles. They influence sperm maturation^13^, sperm viability, capacitation, and acrosome reaction^14^, oocyte maturation^15^, and facilitate the recognition of the conceptus during implantation^16^. Additionally, EVs are involved in critical processes like pregnancy maintenance and parturition^17^. Intriguingly, growing evidence suggests their involvement in pathological conditions such as early pregnancy loss, Polycystic Ovary Syndrome, endometriosis, gestational diabetes mellitus, hypertension, and preeclampsia^18^. This review sheds light on the potential role of EVs in selected aspects of mammalian reproduction.

EVs are known to be involved in intercellular communication in multiple stages of mammalian reproduction. In this communication, we will focus on the EV mediated intercellular communication that occurs between the post fertilization embryo and maternal tissues.

## Embryo-oviduct communication: insights from in vitro models

Understanding the dialogue between embryos and the oviduct is challenging, leading researchers to employ in vitro models to decipher these complex interactions^19^. Utilizing oviductal EVs in embryo culture media has notably enhanced bovine embryos produced in vitro, increasing blastocyst rates, trophectodermal and total cell numbers, and improving cryo-survival post-vitrification^20,21^. Interestingly, EVs enriched using cells collected from different regions of the oviduct exhibit varying impacts on embryonic development; isthmic cell derived EVs were able to enhance survival rates and blastocyst quality, whereas ampullary cell derived EVs show no significant effects^21,22^. Although the precise mechanisms remain elusive, it is hypothesized that the upregulation of the water channel aquaporin 3 (*AQP3*) by the isthmic cell derived EVs might be responsible for the increased survival of blastocysts^22^. Indeed, supplementation of embryo transfer media with oviductal fluid derived EVs in mice significantly increased live birth rates, emphasizing the translational potential of maternal tract EVs in enhancing embryo transfer efficiency in assisted reproduction technologies (ARTs)^23^. Mechanistically, supplemented EVs may induce effects by altering embryonic gene expression leading to altered functional pathways such as protein biosynthesis, nucleotide binding and actin cytoskeleton organization etc^20^. Furthermore, supplementation with frozen-thawed oviductal EVs in bovine embryo culture resulted in differential expression of 221 genes compared to controls, indicating that oviductal EV cargo may mediate effects on embryos through multiple mechanisms, including increased delivery of transcripts, protein translation, and miRNA-based gene silencing^24^.

## Embryonic influence on the oviduct: exploring EV-mediated effects

While extensive research has elucidated the impact of embryos on the oviduct^25–27^, the effects of embryonic EVs on the oviduct have received less attention^28^. Studies have demonstrated that the coculture of bovine embryos with bovine oviductal epithelial cells (BOECs) down-regulates specific genes in the Bone Morphogenetic Protein (BMP) signaling pathway^26^. Recent investigations into embryonic EVs revealed that supplementation with high-quality day 5 embryo-derived EVs altered the expression of 25 genes in BOECs, particularly upregulating interferon-stimulated genes (ISGs) such as *ISG-15, MX1, OAS1Y*, and *LOC100139670*. Notably, interferon-τ (*IFN-τ)*, a type 1 interferon pivotal in pregnancy recognition in ruminants, was enriched in ovine trophoblast and uterine flushing-derived EVs, suggesting a role for EV-mediated communication in conceptus recognition. Additionally, these ISGs were upregulated in oviductal epithelial cells both in vitro and in vivo in the presence of embryos^27,29^, indicating that embryos potentially utilize EVs to inform the mother about their presence or quality. Consequently, further exploration of EV-mediated embryonic effects on the oviduct and oviductal EV effects on embryos is essential to comprehensively understand the mechanisms underpinning embryo-maternal communication in the oviduct.

## Implantation and EV-mediated interactions at the embryo-endometrial interface

Implantation, a pivotal event in early pregnancy, exhibits highly species-specific mechanisms, yet common features exist in successful implantation across species^3,4,30–33^. Notably, bidirectional communication between the embryo and endometrium is a shared characteristic, with emerging evidence implicating EVs in this dynamic process.

Crucial to successful implantation is the recognition of the embryo by the maternal immune system. In ruminants, IFN-τ serves as the primary maternal recognition signal, secreted by the elongating conceptus and acting primarily on the endometrium to inhibit the prostaglandin F2α-mediated luteolytic pathway. Ovine endometrial epithelial EVs are enriched with endogenous retroviral mRNA, which can stimulate *IFN-τ* secretion via Toll-like receptors (TLRs) in the trophectoderm. Similarly, bovine embryonic EVs from uterine flushings, rich in *IFN-τ*, upregulate apoptosis-related genes and adhesion molecules in endometrial epithelial cells, suggesting the involvement of EV-mediated communication in animals with similar placentation patterns^34–37^. In species like pigs, characterized by epitheliochorial placentation without embryo invasion, EVs play a pivotal role in recruiting natural killer (NK) cells and T-cells to the uterine microenvironment, maintaining a proinflammatory status^38^.

One of the key cargo types carried by EVs is miRNAs. Studies have revealed significant differences in miRNA content between serum EV populations from pregnant and non-pregnant domestic animals. For instance, non-pregnant mares exhibited enrichment of miRNAs such as eca-miR-27a, eca-miR-29c, eca-miR-101, and eca-miR-486-5p targeting focal adhesion molecules (FAM), crucial regulators of the extracellular matrix (ECM) and embryo adhesion, indicating their potential as biomarkers of receptivity^39^. In pigs, embryo-derived EVs containing miR-125b induced gene alterations in implantation-linked leukemia inhibitory factor (LIF) and its receptor LIFR in the endometrial epithelium^40^. Conditioned media used in in vitro embryo development are enriched with EVs carrying miRNA cargo that varies with developmental stages. In bovine embryos, EVs are enriched with miRNAs such as miR-24-3p, miR-191, and miR-2887, which influence the endometrial transcriptome and innate immune function^41,42^. These findings underscore the pivotal role of EV-mediated communication in shaping the embryo-endometrial interface during implantation.

## The known unknowns of EV mediated intercellular communication

Even though research into the function of EVs has come a long way since the early days when EVs were considered cellular garbage bags to the current situation where they are recognized as one of the major mediators of intercellular communication, there are still some fundamental questions that remain unanswered regarding the biology of EVs.

## The question of EV uptake and its specificity

The mode EV uptake has been studied intensively during recent years. This uptake involves interactions with recipient cells, altering their physiological or pathological states via surface receptor binding or internalization to release cargo contents. Evidence for internalization includes studies demonstrating direct transfer of mouse RNAs to human mast cells and successful knockdown of gene expression via EV-mediated delivery of siRNAs and luciferin substrates^43,44^. While endocytosis appears as the primary uptake mechanism into the endosomal compartment, the exact pathways—clathrin-mediated endocytosis (CME), caveolin-dependent endocytosis (CDE), micropinocytosis, and phagocytosis—are debated. Tetraspanins, lectins, proteoglycans, and integrins, along with their post-translational modifications, facilitate EV uptake through protein-protein interactions with recipient cell membrane components ^45–48^. Lectins like DC-SIGN and DEC-205 contribute to EV binding and uptake, while tetraspanin-enriched microdomains (TEMs) mediate vesicular fusions^49^. The involvement of tetraspanins and TEMs in EV uptake and cargo delivery is a matter of debate since recent evidence suggests that specific tetraspanins (CD63 and CD9) are not required for membrane fusion or EV uptake^50^. Proteoglycans like Glypican 1 and integrins such as avβ3 and ITGB3 play crucial roles in EV recognition and internalization. Additionally, clathrin and caveolin-mediated endocytosis, macropinocytosis, and phagocytosis processes contribute to EV internalization, each regulated by distinct cellular machinery. Lipid raft involvement in EV uptake is highlighted by their influence on clathrin- and caveolin-mediated endocytosis. Finally, while endocytic mechanisms dominate, direct membrane fusion represents an alternate route for EV uptake involving proteins of Rab family involving a hemi-fusion stalk formation between the lipid bilayers of the EV and the target cell followed by expansion^51^.

Understanding EV uptake specificity remains a key challenge despite advancements in understanding the molecular mechanisms. Studies indicate both broad uptake across cell types and highly specific processes requiring precise surface receptors and ligands for coordinated protein interactions. Modifications in EV surface proteins, like fusion with anti-EGFR nanobodies, alter cell targeting. EVs exhibit cell type-selective uptake and preferential intake by cells of their origin. Factors like recipient cell metabolism and EV characteristics influence uptake, including glycosylation patterns affecting affinity. Heterogeneity in cells and EVs complicates observations. Advancements in technology offer promise for uncovering complex EV uptake mechanisms, showcasing diverse ways cells govern EV-cell communication. At the time of writing, largely due to the studies carried out by researchers such as Bonsergent, Théry, and Lavieu the dogma in the EV field may leaned towards the non-specific EV uptake hypothesis^52–54^. But the functional specificity of EVs is an experimentally established fact. In our own research, we have observed and confirmed that only embryo derived EVs can alter the transcriptomes and proteomes of endometrial cells. EVs deriving from non-embryonic cell lines had little or no effects on the physiology of endometrial cells implying a specificity to the action of trophoblast derived EVs in embryo-maternal communication^3–5,30,33^.

## The question of endosomal escape and cargo delivery

The functional transfer of cargo via EVs hinges on multiple facets encompassing cellular intake, intracellular movement, cargo unloading, and functional effects. Although EV uptake has been extensively studied, our understanding of the molecular mechanisms governing intracellular trafficking and cargo release by EVs is in its infancy.

Following uptake, EVs typically localize within endosomes, with an alternative route being plasma membrane fusion that promptly releases EV content into the cell’s cytosol. Endosomes mature into late stages that eventually merge with lysosomes, but endosomal sorting can also lead to cargo recycling. Such recycling or re-release mechanisms have been observed for EVs. The alteration of recipient cell characteristics by EVs indirectly suggests successful cargo delivery, evading lysosomal degradation.

Several studies indicate that EVs predominantly escape endosomes through membrane fusion, serving as the primary mode of cargo release. Conversely, some research indicates a lack of cargo release due to EVs failing to escape endosomes, which can be overcome by modifying EV surfaces with a fusogenic component like VSV-G^55^. Additionally, alternative pathways such as cargo release via endosomal permeabilization or nuclear import from Rab7+ endosomes through nuclear pores have been proposed^56^.

Despite the insightful research carried out by researchers like refs. ^57,45^ the mechanisms of endosomal escape by EV cargo remains a major hurdle in the pursuit of EV research.

## The question of specific biomolecules responsible for EV mediated communication

Search for biomolecules responsible for EV mediated intercellular communications has always been one of the main driving forces behind EV research. Many biologically active molecules have been demonstrated to be involved with EVs.

Since EVs are especially enriched with regulatory non-coding RNA such as miRNA, it is often hypothesized that EV derived miRNA is responsible for a majority of the transcriptomic, proteomic, and metabolomic alterations observed in target cells after the contact with EVs. There is a considerable body of knowledge connecting specific EV miRNA and their target gene expression in the recipient cells^58,59^. However, balancing these results are the observations that EVs are a very poor source of RNA, with calculated 1 molecule of RNA per 10 EVs and the fact that EV bound RNA species are highly fragmented^60,61^. Taken together with our own observations that show only a minority of transcriptomic alterations can be attributed to EV derived miRNA-based regulation of the recipient transcriptome, leads us to question the potential real-world significance of EV bound small noncoding RNA as a means of intercellular communication. Definitive proof for or against miRNA being a major player in EV bound intercellular communications (especially evidence that carefully considers the quantity of EV miRNA as well as the presence of miRNA in EVs) is, in our opinion, not yet reported.

EVs are enriched with other active biomolecules such as proteins, DNA, and lipids. In case of miRNA not being the principal regulator, one or more of these biomolecules might be the elusive major regulator of EV based intercellular communications. However, the amount of research carried out to investigate the function of these contenders is somewhat less than the EV RNA research.

## The future of EVs in reproductive biology

EVs have emerged as potential players in the landscape of reproductive biology, presenting a promising avenue for transformative discoveries in the near future. Their involvement in intercellular communication within the reproductive system is expected to unveil new dimensions of understanding in gametogenesis, embryogenesis, and various reproductive disorders. EVs are anticipated to serve as diagnostic markers for fertility assessment, especially in the context of non-invasive or minimally invasive embryo quality assays and endometrial receptivity assays^5,14,33,62,63^.

In assisted reproduction, multiple methods are used to assess the quality of spermatozoa, ova, and embryos of all stages of development^5^. Majority of the assessments, based on cellular morphology, morpho kinetics or highly invasive biopsies, do not enjoy a high rate of accuracy^64^. Recent reports suggest that EVs and their cargo could be used as non-invasive markers of gamete and embryo quality. Studies have identified specific biomolecules present in sperm derived EVs associated with sperm motility, morphology, and DNA integrity^65,66^. Furthermore, alterations in the EV profile have been correlated with male infertility and reproductive disorders^67,68^. Similarly, specific proteins present in oocyte derived EVs are associated with oocyte maturation, fertilization potential, and embryo development^69,70^. These EV based biomarkers could be developed into non-invasive diagnostic assays to measure the gamete quality.

In the infancy of assisted reproduction, multiple embryo transfer was the norm. The current practice is changing in favor of elective single embryo transfer or elective dual embryo transfer due to their higher success rates. Embryo selection is a requirement in elective practices and the methods used to select the embryos are thought to play a major role in the success of the transfer^71–73^. There are reports that suggest that embryo quality could be measured with a high accuracy using embryo derived EVs (EV concentration^74^ and the contents of EVs) and the effects of embryo derived EVs on the maternal cells^62^. Once fully optimized and developed, these EV bound biomarker-based diagnostics will revolutionize the field of assisted reproduction, increasing the success rate of embryo transfers and reducing the mental and physical discomfort of patients.

EVs could offer novel therapeutic opportunities for infertility treatments. Multiple reports suggest that EVs from various sources such as pluripotent stem cells and mesenchymal stem cells could be used as therapeutic agents in infertility related conditions such as primary ovarian insufficiency and Infertility with intrauterine adhesions^75–77^. Efforts are being taken to bring these therapeutic agents to patient use via clinical trials^78,79^. With adequate clinical evidence and proper regulations, these EV based therapies will be able to help patients with infertility issues in the future.

The integration of EV research with the concept of One Health is poised to revolutionize our comprehension of reproductive health. Understanding how environmental factors, such as pollutants or toxins, influence EV composition and function in the reproductive system could unveil critical links between environmental exposures and fertility outcomes. These insights could pave the way for the development of biomarkers indicating reproductive health risks posed by environmental pollutants, thus advancing preventive strategies and interventions to mitigate the adverse effects of environmental factors on fertility.

The future of EVs in reproductive biology is entwined with multidisciplinary collaborations, combining expertise from molecular biology, reproductive sciences, environmental health, and clinical medicine. By unraveling the enigmatic roles of EVs in reproductive processes, we are poised to unlock a trove of insights that will not only revolutionize fertility treatments and diagnostics but also foster a deeper understanding of the interconnectedness between environmental factors, reproductive health, and the broader concept of One Health (Box 1).

Box 1 The status quo, the unknowns and the future directions of EV mediated intercellular communication in human reproduction **Important developments**

Recognition of EVs as significant mediators in embryo-maternal communication during reproduction.

EVs’ diverse roles in sperm and oocyte maturation, embryo development, implantation, and pregnancy maintenance, along with their implications in reproductive pathologies.

**Outstanding questions**

Understanding EV uptake specificity and the mechanisms governing cargo delivery to recipient cells.

Identifying the precise biomolecules responsible for EV-mediated intercellular communication, especially regarding EV RNA efficacy and roles of proteins, DNA, and lipids.

**Technical challenges**

Unraveling the complexities of EV uptake mechanisms, including cell-type specificity and the involvement of surface proteins.

Investigating endosomal escape of EV cargo.

Deciphering the specific molecules driving functional effects.

**Future directions**

Potential applications of EVs as diagnostic biomarkers for fertility assessment and targeted drug delivery in reproductive tissues.

Exploring EVs' role in environmental health and their link to fertility outcomes for preventive interventions.

Utilizing EV research to improve prenatal care, maternal outcomes, and personalized medicine in reproductive health.

Leveraging multidisciplinary collaborations to uncover the intricate roles of EVs in reproductive biology and broader health contexts.

## Data Availability

The data that support the conclusions of this review are openly available in the published literature and relevant online repositories. All sources cited in this paper are appropriately referenced. No new data was generated for this review.
